# Impaired Remodeling of Tight Junctions Associated with Loss of Enamel Rod Decussation in *Tgfbr2*^*G357W/+*^ Mice

**DOI:** 10.1007/s00223-026-01541-7

**Published:** 2026-04-30

**Authors:** Olivier Duverger, Valentina Baena, Zulfeqhar A. Syed, Janice S. Lee

**Affiliations:** 1 Craniofacial Anomalies and Regeneration Section, National Institute of Craniofacial and Dental Research, National Institutes of Health, Bethesda, MD USA; 2 Electron Microscopy Core, National Heart Lung and Blood Institute, National Institutes of Health, Bethesda, MD USA

**Keywords:** Loeys-dietz syndrome, *TGFBR2*, Enamel, Rod decussation, Ameloblasts, Tight junctions

## Abstract

During enamel secretion, ameloblasts follow a complex coordinated movement that results in the enamel rods being arranged in a crisscross pattern that contributes significantly to its exceptional mechanical properties. We previously determined that mutations in the gene encoding TGF-β receptor 2 (TGFR-2) in humans (*TGFBR2*) and mice (*Tgfbr2*) result in the formation of fully mineralized enamel that exhibits impaired decussation of enamel rods. Using *Tgfbr2*^*G357W/+*^ mice, we demonstrated that this phenotype was due to disrupted coordinated movement of ameloblasts during enamel secretion. In this study, we used focused ion beam scanning electron microscopy (FIB-SEM) to look more closely at the area of the apical terminal web (ATW) where the cells are connected via tight junctions. We show that ameloblasts from wild-type littermates exhibit an abrupt shift in orientation at the ATW while *Tgfbr2*^*G357W/+*^ mice show no signs of such shift. Moreover, ZO-1 distribution at the ATW was abnormal in *Tgfbr2*^*G357W/+*^ mice with reduced accumulation at the ATW and presence of clusters of the protein at the basal side. These results suggest that abrupt orientation shift at the ATW via dynamic remodeling of tight junctions is essential for rows of ameloblasts to deposit enamel rods in opposite directions.

**Supplementary Information:**

The online version contains supplementary material available at 10.1007/s00223-026-01541-7.

## Introduction

The exceptional mechanical properties of dental enamel are due not only to its high mineral content (96% hydroxyapatite) but also to the complex crisscross arrangement of enamel rods, known as enamel rod decussation [1–5]. This rod pattern is the result of a tightly controlled movement of ameloblasts (enamel-producing cells) during enamel secretion, a complex developmental process that dentists, biologists and material scientists have observed, modeled and tried to elucidate for over a century [1–5]. Up to recently, loss of enamel rod decussation in disease and animal models had only been observed in association with severe hypomineralization of enamel, like in response to deletion of the genes encoding the metalloprotease MMP20 [6], the transcription factor DLX3 [7], or the multi-functional proteinase ADAM10 [8]. We recently described a disease and animal model in which ameloblasts were fully functional in their ability to deposit the enamel matrix and to fully mineralize it but failed to follow the coordinated movement that leads to rod decussation, resulting in abnormal ultrastructure and impaired mechanical properties [9]. This phenotype is the result of autosomal dominant mutations in the gene encoding transforming growth factor beta receptor 2 (*TGFBR2*) that cause Loeys-Dietz syndrome 2 (LDS2; M.I.M. 610168). Our previous work using a heterozygous mouse model for LDS2 (*Tgfbr2*^*G357W/+*^ mice) has elucidated part of the molecular changes that are associated with this phenotype, including an increase in the activity of the metastasis suppressor NDRG1, decreased activity of Rac1/Cdc42 and decreased activity of myosin II at the apical terminal web (ATW) [9]. These changes are consistent with a disruption of the ability of ameloblasts to rearrange their cytoskeleton and cell-cell interactions to allow for proper cell movement.

In this study, we take a closer look at the architecture of the tight junctions at the ATW and analyze the expression pattern of ZO-1, a key player in tight junction assembly, in ameloblasts from *Tgfbr2*^*+/+*^ and *Tgfbr2*^*G357W/+*^ mice.

## Materials and Methods

### Animals

*Tgfbr2*^*G357W/+*^ mice (*129(Cg)-Tgfbr2*^*tm1.1Hcd*^*/J*) were purchased from the Jackson Laboratories (Bar Harbor, ME) [10]. The mice were maintained by mating heterozygous mice with wild-type 129 mice to produce heterozygotes and wild-type littermates that were used as control for all experiments. Single housed mice were provided with environmental enrichment (hut). All animal work was part of an animal study protocol that was approved by the NIDCR Animal Care and Use Committee.

### Focused Ion Beam Scanning Electron Microscopy (FIB-SEM)

Mice at P18 were anesthetized and perfused with 10 ml of 0.1% sodium cacodylate buffer pH 7.4 followed by 10 ml of fixative solution containing 2.5% glutaraldehyde and 1% paraformaldehyde in 0.1 M sodium cacodylate buffer, pH 7.4. After perfusion, the mandibles were dissected and incubated in fixative solution for two hours at room temperature followed by additional overnight incubation at 4 °C with gentle agitation.

After fixation, the mandibles were washed with 0.1 M sodium cacodylate buffer 5 times for 5 min each. They were then incubated in 2% osmium tetroxide in 1.5% potassium ferricyanide (Electron Microscopy Sciences, Hatfield, PA) made in 0.1 M sodium cacodylate buffer for 1 h on ice, followed by 5 water washes for 5 min each. All subsequent water washes mentioned here consist of 5 washes for 5 min each. The mandibles were incubated in 1% thiocarbohydrazide (Electron Microscopy Sciences, Hatfield, PA) in water for 20 min at room temperature followed by water washes. This was followed by an incubation in 2% osmium tetroxide in water for 30 min on ice and subsequent water washes at room temperature. Mandibles were then incubated in 1% uranyl acetate (Electron Microscopy Sciences, Hatfield, PA) in water overnight at 4 °C. The following day, the mandibles were washed in water and then placed in a solution of 0.066% lead aspartate for 30 min at 60 °C followed by water washes at room temperature. The mandibles were dehydrated with a series of graded ethanol in water incubations consisting of 30%, 50%, 70%, 90%, three 100% ethanol, and two with propylene oxide (Electron Microscopy Sciences, Hatfield, PA) for 10 min each. This was followed by infiltration with Epon resin (Embed 812, Electron Microscopy Sciences, Hatfield, PA) using increasing concentrations of resin dissolved in propylene oxide over the course of 48 h. The mandibles were then mounted on their side on SEM stubs with fresh Epon resin and cured in a 60 °C oven for two days.

After polymerization, the incisor was polished along the cervical-incisal axis using a diamond trimming knife until the middle region of the incisor was exposed as judged by toluidine blue staining. The region of interest was selected midway between the initiation of enamel secretion and the transition zone that precedes the maturation zone, clearly distinguishable based on the development of the papillary layer [9]. The sample was then coated with a 40 nm-thick layer of gold using a sputter coater (Electron Microscopy Sciences, Hatfield, PA). The region of interest for focused ion beam scanning electron microscopy (FIB-SEM) imaging was determined after SEM imaging of the exposed cross-section of the incisor using a SESI detector (Carl Zeiss Microscopy, White Plains, NY). FIB-SEM imaging was carried out with a Crossbeam 540 (Carl Zeiss Microscopy, White Plains, NY) using Atlas 5 (Fibics Inc., Ottawa, Canada). FIB-SEM images were acquired at 6 nm in XY and a slice thickness of 12 nm. The SEM beam was used at 1.5 kV and 2 nA, and the FIB was used at 30 kV. Images were acquired with the enhanced back-scatter detector with a grid voltage set to 500 V. After the volume acquisition, the image contrast was inverted, the stack was registered using the Atlas 5 registration module, and the X and Y pixels were binned by 2 to create an isotropic volume. Videos and screenshots of the volume were taken with Imaris (Oxford Instruments, Abingdon, UK).

### Segmentation of FIB-SEM Datasets and 3D Rendering

Volume rendering was generated to visualize the change in the relative position of adjacent rows of ameloblasts at the ATW. A deep learning-based segmentation pipeline was implemented in python using a U-Net architecture with ResNet34 encoder constructed with segmentation-models library. Ground truth annotations were manually generated using the Labkit plugin in ImageJ. High resolution grayscale training images and corresponding masks were normalized and divided into 256 × 256 pixel patches for training. The model was trained using binary cross-entropy loss and the Adam optimizer for 100 epochs. Segmentation was performed slice-by-slice and after inference, probability maps were binarized using a threshold of 0.5. After manual inspection, final binary masks were reconstructed into 3D volumes using Imaris (v9.9). Six cells from two adjacent rows of ameloblasts (three cells for each row) were segmented by erasing the mask of all the other cells in each of 200 slices covering the ATW.

### Immunohistochemical Analysis

Specimens were fixed in 4% PFA in 1X PBS overnight, washed three times in 1X PBS, decalcified using 10% EDTA solution for two weeks (solution replaced every 2 to 3 days), and embedded in paraffin using standard method. 6 μm sections were made and placed on Superfrost(R)→ Plus slides (VWR, Radnor, PA).

For immunohistochemical staining, tissue sections were deparaffinized in xylene (twice) and rehydrated through and ethanol series (100% twice, 95%, 70%, water). Antigen retrieval was performed using citric acid solution (Vector Laboratories, Newark, CA). Sections were incubated in a block solution containing 5% goat serum (or donkey serum for goat antibodies) and 7.5% BlokHen II™ (Aves Labs, Tigard, OR) in 1X PBS. Primary antibody used: rabbit anti-ZO-1 (Thermo Fisher Scientific, Waltham, MA). Secondary antibody used: goat anti-rabbit Alexa Fluor 555 (Thermo Fisher Scientific, Waltham, MA). Nuclei were stained using DAPI (Thermo Fisher Scientific, Waltham, MA). Actin filaments were stained using Phalloidin Alexa Fluor 647 (Thermo Fisher Scientific, Waltham, MA). Fluorescent confocal images were acquired on a Leica SP8 instrument (Leica Microsystems, Wetzlar, Germany).

## Results

### Secretory Ameloblasts in Wild-type Mice Exhibit an Abrupt Shift in Cell Orientation at the Apical Terminal Web, Aligned with Rod Decussation

**Fig. 1:**
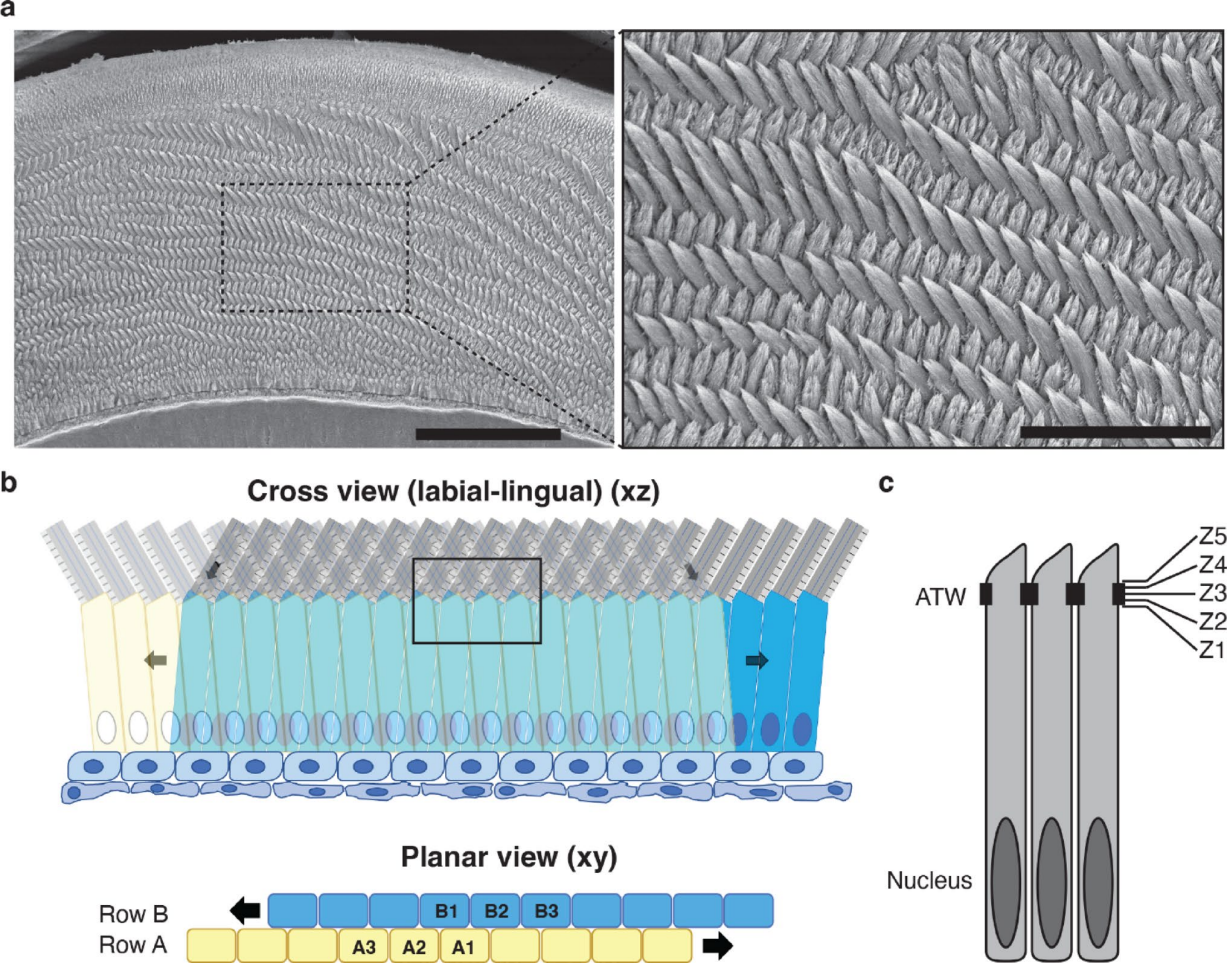
Enamel rod decussation, ameloblast movement during enamel secretion, and apical terminal web. **a** Scanning electron microscopy image of polished cross section of mouse continuously growing incisors highlighting enamel rod decussation (crisscross pattern). Scale bars: 50 μm for the left panel, 20 μm for the magnified inset on the right. **b** Schematic representation of the alternating orientation of enamel rod deposition in the mouse continuously growing incisor. The cross-section view (xz plane) is shown at the top, and the planar view (xy plane) is shown at the bottom. Rows of cells depositing enamel in alternating orientations are depicted in different colors: cells in rows A (yellow) are leaning to the right, whereas cells in rows B (blue) are leaning to the left, as they deposit enamel. The black rectangle marks the region of interest selected at the interphase between the ameloblasts and the enamel matrix for focused ion beam scanning electron microscopy (FIB-SEM) imaging. **c** Schematic representation of three ameloblasts highlighting the tight junctions (black boxes) at the apical terminal web (ATW). The five marked positions (z1-5) indicate the position of the slices that were analyzed from the FIB-SEM dataset. 201 slices (between z1 and z5) covering the area of the ATW were selected. z1 = slice 1; z2 = slice 51; z3 = slice 101; z4 = slice 151; z5 = slice 201Enamel rod decussation, ameloblast movement and apical terminal web.

During enamel formation, enamel rods are deposited following an intricate crisscross pattern through a process known as enamel rod decussation. In rodent continuously growing incisors, this pattern is clearly noticed on cross sections of the teeth showing alternating orientation of the rods from one row to the next (Fig. 1a). The schematics in Fig. 1b shows how ameloblasts in mouse continuously growing incisors (cross section) deposit enamel at an angle that shifts from one row of ameloblasts to the next. In our previous report, using focused-ion-beam scanning electron microscopy (FIB-SEM) at the interphase between the enamel matrix and ameloblasts (Fig. 1b, black rectangle), we were able to trace the “trail” left by ameloblast as they secreted enamel and show that ameloblasts deposit enamel in alternating orientations from one row to the next [9].

Here, we focused on the area of the ATW, where tight junctions provide a clear visualization of the separation between the cells that exhibit a cobblestone pattern. We selected 200 slices starting just before the appearance of the tight junctions on the most proximal side of the ATW (rough endoplasmic reticulum) and ending right after the tight junctions on the distal side (near the Tomes’ processes and the enamel matrix) (Fig. 1c).

**Fig. 2:**
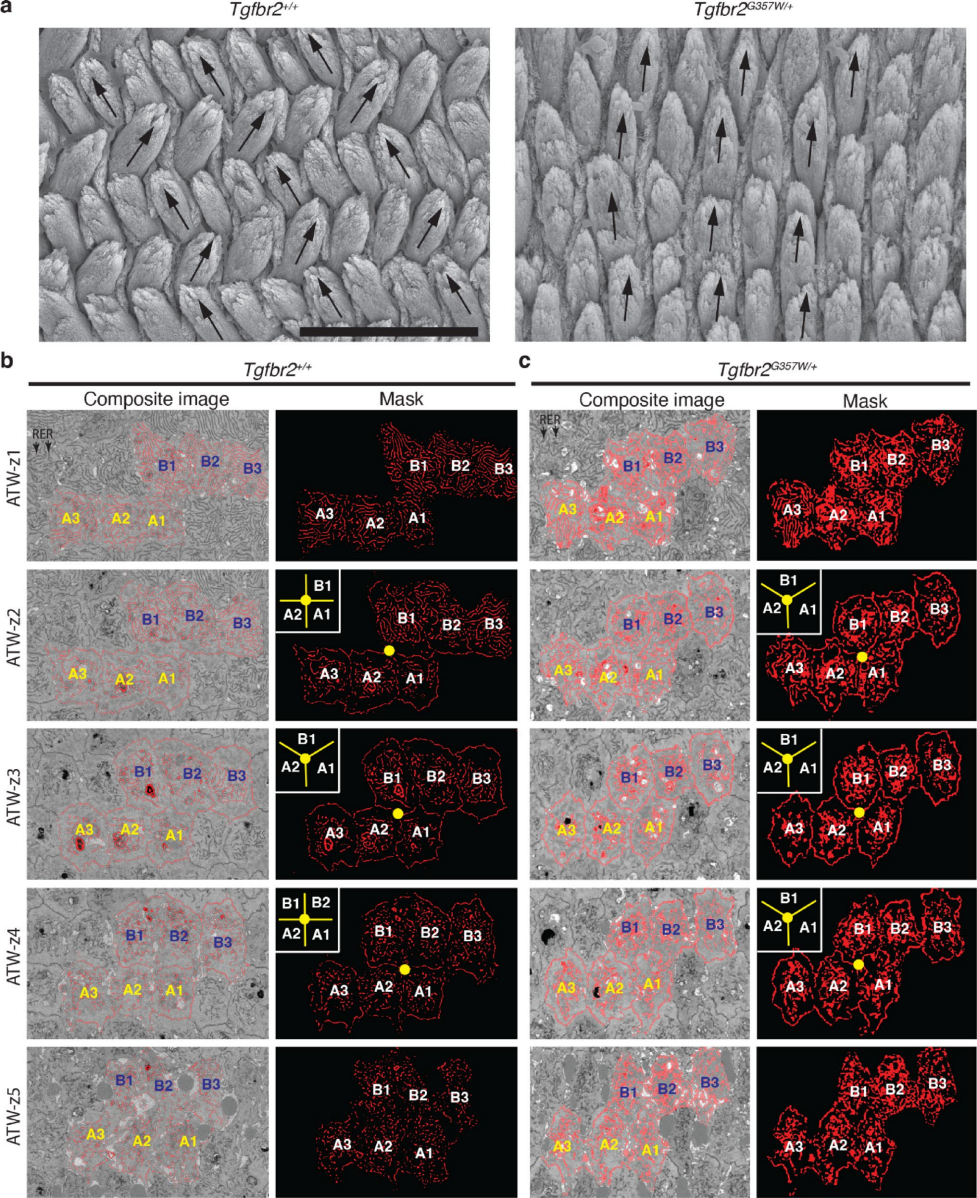
Loss of abrupt shift in secretory ameloblast orientation at the apical terminal web in *Tgfbr2*^*G357W/+*^mice. **a** Scanning electron microscopy images of polished cross sections of continuously growing incisors from *Tgfbr2*^*+/+*^ and *Tgfbr2*^*G357W/+*^ mice showing the disruption of enamel rod decussation (crisscross pattern) in *Tgfbr2*^*G357W/+*^ mice. Black arrows highlight the change of enamel rod orientation from one row of enamel rods to the next in *Tgfbr2*^*+/+*^ mice, but not in *Tgfbr2*^*G357W/+*^ mice. Scale bar: 10 μm. **b and c** Five FIB-SEM slices showing the relative position of six cells segmented from two adjacent rows (three per row) as we progress from the proximal (z1; RER, rough endoplasmic reticulum) to the distal part (z5) of the ATW (see Fig. 1c), in ameloblasts from *Tgfbr2*^*+/+*^ (**b**) and *Tgfbr2*^*G357W/+*^ mice (**c**). Left panels show composite images that include the raw image and the mask obtained after segmentation of the six cells. Right panels show the mask alone for better visualization. Note the abrupt change in the relative position of the cells from row A and row B in *Tgfbr2*^*+/+*^ mice (**b**). The yellow dot on the mask image marks the intersection point between cells A1 and A2, and cells in row B. The insets in the upper left corner show the shift in cell position around this intersection point, as we go from z2 to z4 (clear tight junctions). See Suppl Movies 1 and 2 for dynamic visualization of all the slices. Note that the relative positions of the cells from row A and row B remain unchanged through the whole ATW region for *Tgfbr2*^*G357W/+*^ mice (**c**). The yellow point and insets in the upper left corner show that the cells stay in a static position around the intersection point as we go from z2 to z4. See Suppl Movies 3 and 4 for dynamic visualization of all the slices.

As we previously reported, heterozygous mutation in the *Tgfbr2* gene (*Tgfbr2*^*G357W/+*^ mice) results in loss of enamel rod decussation, with no significant change in enamel volume and mineral density [9]. In the present study, we used this animal model to analyze the structure of tight junctions at the ATW in mice showing normal rod decussation (*Tgfbr2*^*+/+*^ mice) and in mice failing to create the characteristic decussation of enamel rods (Fig. 2a). As shown in Fig. 2b and Suppl Movies 1 and 2, ameloblast in wild-type mice (*Tgfbr2*^*+/+*^ mice) exhibited an abrupt shift in cell direction at the ATW, in the direction of rod decussation.

Segmentation of six cells from two adjacent rows (three cells from each row) allowed to appreciate this shift more clearly (Fig. 2b). Numbering of the three cells in row A as A1, A2 and A3 (A1 leading the front of the shift), and the three cells in row B as B1, B2 and B3 (B1 leading the front of the shift), we could appreciate that, although A1 and B1 were the only cells that exhibited extensive cell-cell contact on the proximal side of the ATW, all three cells in row A ended up being in direct contact with a cell in row B (A1-B3, A2-B2 and A3-B1) on the distal side of the ATW, a position at which A1 and B1 were no longer in direct contact (Fig. 2b).

These results indicate that ameloblasts undergo dynamic remodeling of their tight junctions at the ATW to allow for the cells in adjacent rows to slide against one another and pursue their own path in opposite directions, resulting in rod decussation.

### Secretory Ameloblasts in *Tgfbr2*^*G357W/+*^ Mice Fail to Shift their Orientation at the Apical Terminal Web

FIB-SEM analysis performed on ameloblasts from *Tgfbr2*^*G357W/+*^ mice revealed that the abrupt shift in the direction of ameloblasts at the ATW was not observed. Although the cells exhibited clear tight junctions and a normal cobblestone pattern, the relative position of the cells from two adjacent rows remained the same at the most proximal and the most distal part of the ATW (Fig. 2c).

Segmentation of six cells from two adjacent rows (three cells for each row) allowed to appreciate this more clearly (Fig. 2c and Suppl Movies 3 and 4). Contrary to what was observed in *Tgfbr2*^*+/+*^ mice, the relative position of the cells in row A (A1, A2 and A3) to the cells in row B (B1, B2 and B3) did not change between the proximal and distal sides of the tight junctions at the ATW in *Tgfbr2*^*G357W/+*^ mice (Fig. 2c).

**Fig. 3:**
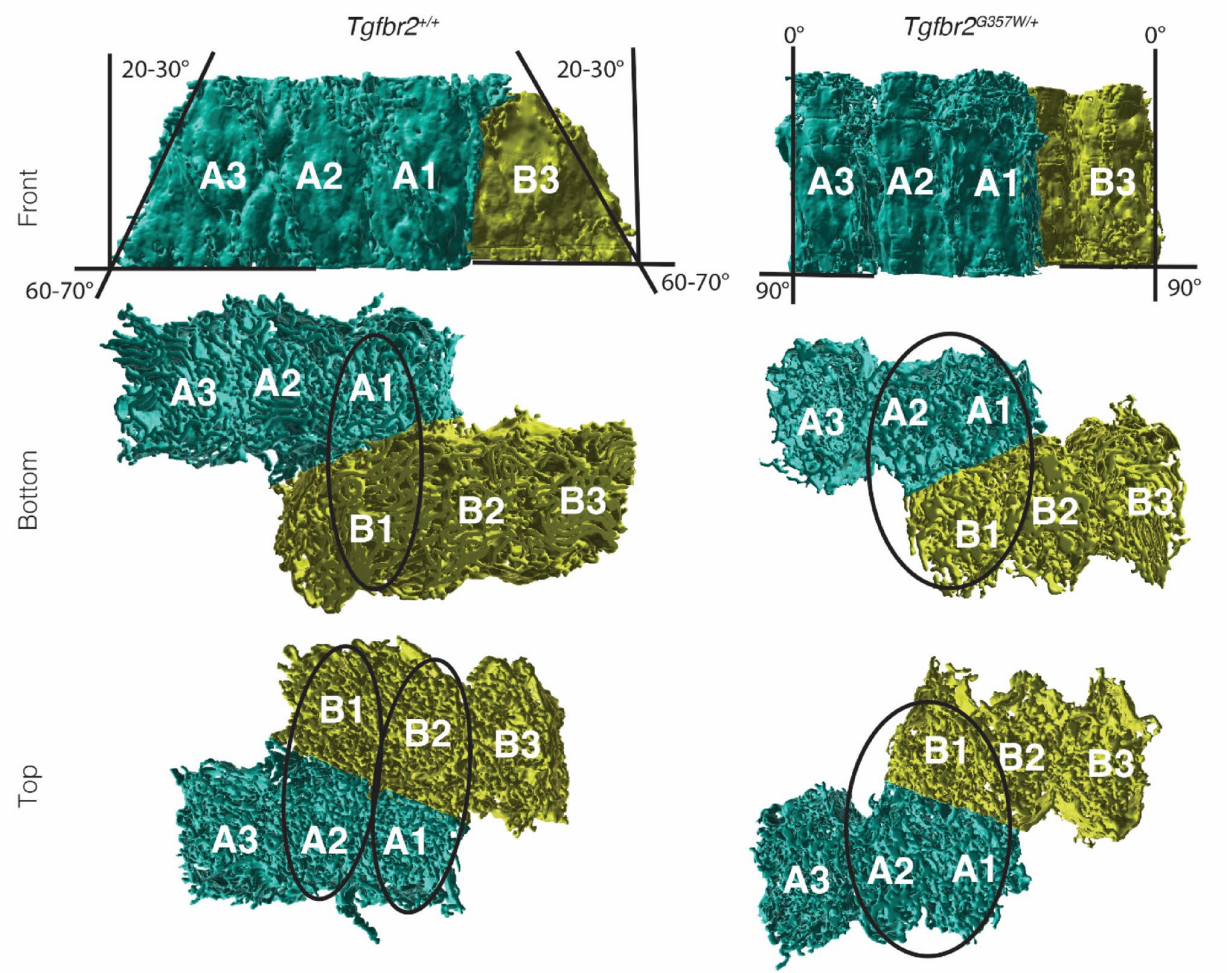
3D visualization of the ATW region in *Tgfbr2*^*+/+*^ and *Tgfbr2*^*G357W/+*^ mice. 3D reconstruction and visualization of the apical terminal web (ATW) of the six segmented cells from two adjacent rows of ameloblasts in *Tgfbr2*^*+/+*^ and *Tgfbr2*^*G357W/+*^ mice. Front, top and bottom views are shown. Note that the ATWs in *Tgfbr2*^*+/+*^ mice are tilted at opposite angles in row A and row B, while they are all vertically oriented in *Tgfbr2*^*G357W/+*^ mice. Black lines have been added to the front view to mark the 20–30° angle formed between the vertical plane and the orientation of the ATW in *Tgfbr2*^*+/+*^ mice. This angle is null in *Tgfbr2*^*G357W/+*^ mice. Ellipses for *Tgfbr2*^*+/+*^ mice highlight the interaction between A1 and B1 on the proximal side of the ATW (bottom view), while the distal side of the ATW (top view) shows interaction between A1 and B2, and between A2 and B1. The ellipses for *Tgfbr2*^*G357W/+*^ mice highlight the interaction between A1, A2 and B1, both on the proximal (bottom view) and the distal (top view) sides of the ATW

Through 3D reconstruction of the six selected cells, we were able to visualize this phenotype in 3D. While the reconstruction obtained from the *Tgfbr2*^*+/+*^ dataset shows two rows of ATWs oriented in opposite directions and at an approximate angle of 20–30° from a vertical reference line, the reconstruction obtained from the *Tgfbr2*^*G357W/+*^ dataset shows six perfectly vertical ATWs (Fig. 3 and Suppl Movies 5 and 6).

These results indicate that ameloblasts in *Tgfbr2*^*G357W/+*^ mice fail to undergo the sharp shift at the ATW observed in ameloblasts from wild-type mice. This suggests that ameloblast are unable to rearrange their tight junctions at the ATW to allow for this shift to happen, therefore resulting in failure to deposit enamel rods in opposite directions.

### The Tight Junction Protein ZO-1 Exhibits a Disrupted Distribution in Ameloblasts in *Tgfbr2*^*G357W/+*^ Mice

**Fig. 4:**
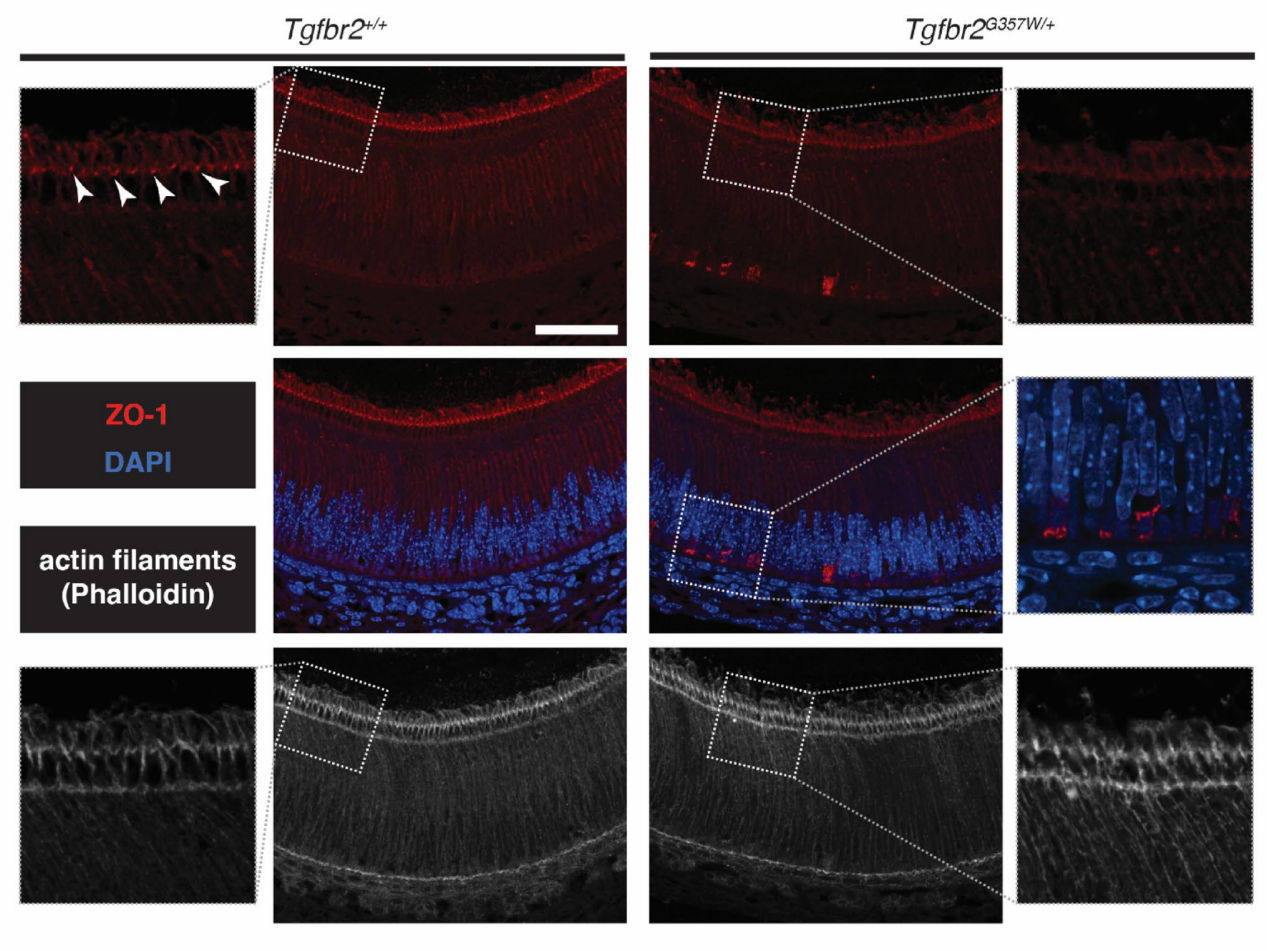
Altered distribution and aberrant clustering of ZO-1 in ameloblasts from *Tgfbr2*^*G357W/+*^ mice. Immunohistochemical analysis of ZO-1 (Alexa-555, red) distribution in secretory ameloblasts from *Tgfbr2*^*+/+*^ and *Tgfbr2*^*G357W/+*^ mice. DAPI was used to stain the nuclei (blue). Note the reduced accumulation at the apical terminal web (white arrowheads) and the presence of clusters on the basal side of the cells in *Tgfbr2*^*G357W/+*^ mice. Lower panels show the distribution of actin filaments stained with Phalloidin Alexa 647 (gray). Scale bar: 50 μm

Our FIB-SEM observations, together with our previous findings that ameloblasts in *Tgfbr2*^*G357W/+*^ mice are functional in their ability to produce fully mineralized enamel, indicate that tight junctions are structured normally and create the proper epithelial barrier. However, they are static and fail to be remodeled to allow ameloblasts to move, consistent with reduced myosin II activity at the ATW [9]. Zonula-occludens-1 (ZO-1) is a key scaffolding protein in tight junction remodeling, linking transmembrane proteins to the actin cytoskeleton [11]. Immunodetection of ZO-1 in ameloblasts indicated accumulation of the protein at the ATW in *Tgfbr2*^*+/+*^ mice (Fig. 4). This distribution was altered in *Tgfbr2*^*G357W/+*^ mice, with reduced accumulation at the ATW and presence of clusters of ZO-1 on the basal side of the cells (Fig. 4). Despite possible alterations in fine architecture, the accumulation of actin filaments at the ATW was still clearly visible in *Tgfbr2*^*G357W/+*^ mice.

These results indicate that alteration in ZO-1 regulation at the ATW, likely related to decreased activation of myosin II, is involved in the defective remodeling of ameloblast tight junctions in *Tgfbr2*^*G357W/+*^ mice.

## Discussion

Amelogenesis is a complex process through which ameloblasts deposit the enamel matrix in contact with the developing dentin [12]. As ameloblasts go through the secretion and the maturation stage, they need to keep the epithelial barrier intact to allow for a tight control of both transcellular and paracellular transport of fluids and ions between the circulation (vascularized underlying tissues) and the enamel space [13, 14]. Paracellular transport is controlled by tight junctions that regulate the diffusion of fluids and minerals between adjacent cells. However, during the secretion stage, ameloblasts follow a complex coordinated movement as they deposit enamel to drive the decussation of enamel rods. This process needs to happen all while maintaining the epithelial barrier, which means that tight junctions need to be constantly remodeled to allow for adjacent cells to move in different directions.

In this study, we show for the first time that secretory ameloblasts exhibit an abrupt shift in cell orientation right around the ATW where ameloblasts are connected by tight junctions. Using a model in which ameloblasts can produce fully mineralized enamel but fail to produce the characteristic decussation pattern of enamel rod, we show that this abrupt shift at the ATW does not happen. These findings suggest that this localized shift is essential for the ability of rows of ameloblasts to “slide” in opposite directions as they deposit enamel rods.

Consistent with these observations, we previously determined that secretory ameloblast in *Tgfbr2*^*G357W/+*^ mice exhibited reduced myosin II activity at the ATW, a process that appears to involve a reduction in Rac1/Cdc42 activity [9]. Here we show that the distribution of the tight junction protein ZO-1, which is also regulated by Rac1/Cdc42 [15, 16] and by myosin II activity [17], is disrupted, with reduced accumulation at the ATW and aberrant formation of clusters at the base of the cells.

**Fig. 5:**
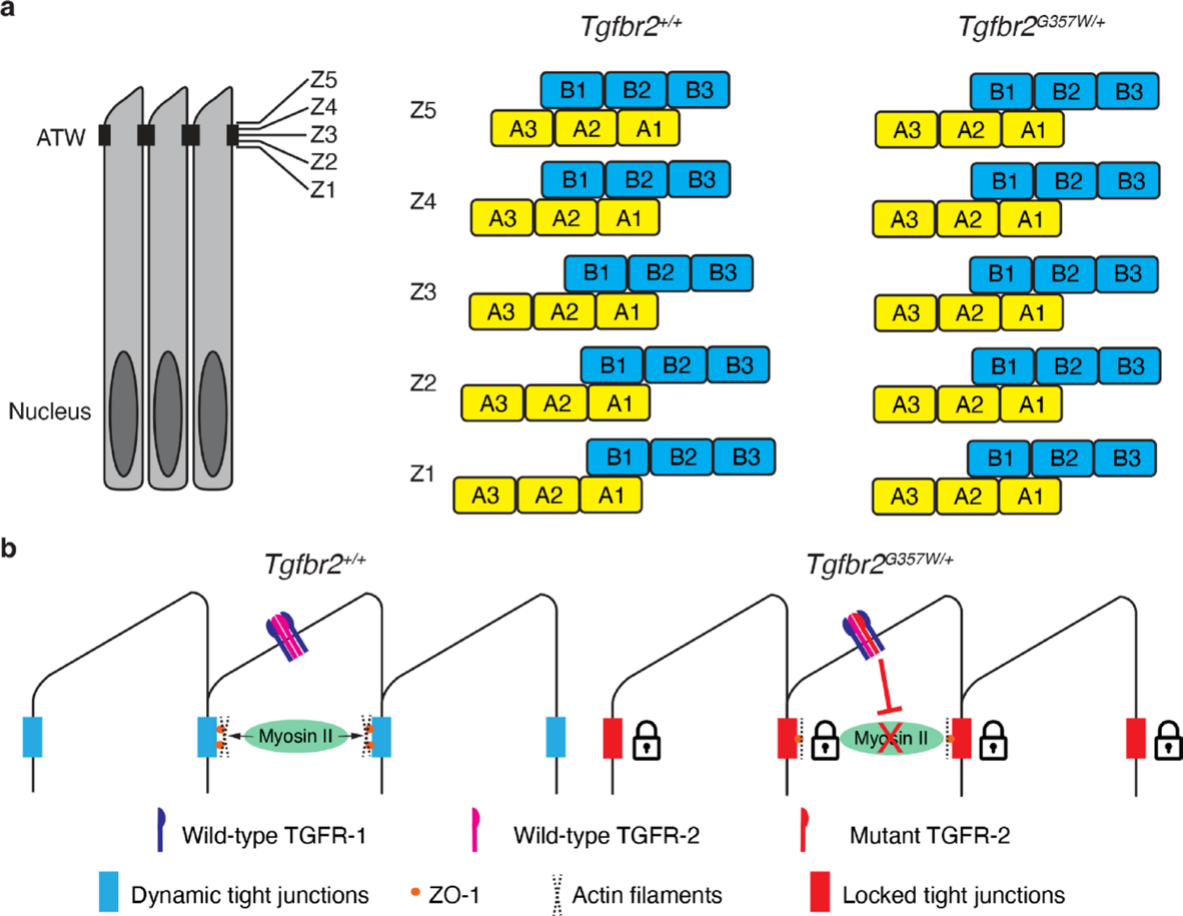
Schematic model showing the disruptive effect of mutation in TGFR-2 on the remodeling of tight junctions at the apical terminal web. **a** Schematic representation of the relative movement of cells from row A and row B, from the proximal part (z1) to the distal part (z5) of the ATW in *Tgfbr2*^*+/+*^ and *Tgfbr2*^*G357W/+*^ mice. **b** Schematic representation of tight junctions between ameloblasts being remodeled through myosin II activity to allow ameloblast to lay enamel rods in opposite directions as they migrate away from the matrix they deposit. Mutation in TGFR-2 results in tight junctions that are locked in place due to disruption of myosin II activity and ZO-1 distribution at the ATW, making it impossible for ameloblasts to move in opposite directions to create the decussation pattern of enamel rods

Taken together, these findings indicate that ameloblasts in *Tgfbr2*^*G357W/+*^ mice are unable to remodel their tight junctions to allow for coordinated movement of the cells during enamel rod deposition (Fig. 5a). Through a mechanism that we have only partially elucidated, mutations in TGFR-2 result in disruption of myosin II activity and ZO-1 regulation at the apical terminal web, causing the tight junctions to be locked in place and unable to be remodeled for proper directional movement of ameloblast (Fig. 5b). LDS2 is so far the only disease model showing structural enamel anomalies with no change in enamel volume and mineral density, which makes it a unique tool to understand the mechanism that controls ameloblast movement during enamel secretion. We anticipate that more disease and/or mouse models with a similar phenotype will be discovered in the future.

## Supplementary Information

Below is the link to the electronic supplementary material.

Supplementary Material 1

Supplementary Material 2

Supplementary Material 3

Supplementary Material 4

Supplementary Material 5

Supplementary Material 6
